# Evaluation of the Diagnostic Role of Bedside Lung Ultrasonography in Patients with Suspected Pulmonary Embolism in the Emergency Department

**DOI:** 10.4274/balkanmedj.2016.1181

**Published:** 2017-08-04

**Authors:** Hüseyin Acar, Serkan Yılmaz, Elif Yaka, Nurettin Özgür Doğan, Asım Enes Özbek, Murat Pekdemir

**Affiliations:** 1 Clinic of Emergency, Tunceli State Hospital, Tunceli, Turkey; 2 Department of Emergency, Kocaeli University School of Medicine, Kocaeli, Turkey; 3 Clinic of Emergency, Derince Training and Research Hospital, Kocaeli, Turkey

**Keywords:** Pulmonary embolism, ultrasonography, diagnostic ultrasound, emergency departments, bedside testing

## Abstract

**Background:**

Despite the existence of detailed consensus guidelines, challenges remain regarding efficient, appropriate, and safe imaging methods for the diagnosis of suspected pulmonary embolism.

**Aims:**

To investigate the role of the wedge sign, B-lines, and pleural effusion seen on bedside lung ultrasound in the diagnosis of pulmonary embolism.

**Study Design:**

Diagnostic accuracy study.

**Methods:**

During the first evaluation of patients with suspected pulmonary embolism, bedside lung ultrasound was performed, and the B-lines, wedge sign, and pleural effusion were investigated. Computed tomography angiography was used as a confirmatory test and was compared with the lung ultrasound findings.

**Results:**

Pulmonary embolism was detected in 38 (38%) patients. In the comparison of bedside lung ultrasound results, statistically significant differences were found between the groups in terms of the B-lines and wedge sign (p=0.005 and p<0.001, respectively). There were no significant differences in terms of effusion (p=0.234). Comparison of these findings with computed tomography angiography of the chest showed weak negative correlations between the groups in terms of B-lines (r=-0297) and a moderately positive correlation in terms of the wedge sign (r=0.523). The sensitivity, specificity, and positive and negative predictive values of lung ultrasound findings alone were low. In the logistic regression analysis, the wedge sign (p<0.01, OR=69.45, 95% CI=6.94-695.17) and B-line (p=0.033, OR=1.96, 95% CI=0.41-8.40) were found to be effective in the diagnosis of pulmonary embolism.

**Conclusion:**

Although the role of lung ultrasound has been increasing in the management of critically ill patients, its value is limited and cannot replace the gold standard tests in the diagnosis of pulmonary embolism.

Pulmonary embolism (PE) is a disease with a high mortality rate. The diagnosis of PE is difficult because the signs and symptoms may be nonspecific. Imaging methods, such as ventilation/perfusion scintigraphy, pulmonary angiography, and spiral computed tomography (CT), which evaluate pulmonary circulation, are used in the diagnosis of PE, but challenges still exist (1,2).

In recent years, bedside lung ultrasonography (LUS) has been used as a diagnostic tool to complement traditional radiographic methods in the diagnosis of a variety of mediastinal and pleural conditions, as well as for detecting pleural effusions and as a guide in pulmonary thoracentesis (1). LUS has been increasingly considered to be a quick and safe method in the diagnosis of pneumothorax, pleural effusion, pulmonary oedema, and pneumonia (3). Lichtenstein and Mezière (4) (2008) developed the Bedside Lung Ultrasound in Emergency (BLUE) protocol, an algorithm that identifies the diagnostic role of LUS in demonstrating the many serious conditions that cause acute respiratory failure.

Visualisation of thromboembolic pulmonary lesions by sonography was first described in the late 1960s. Joyner et al. (5) (1966) reported that LUS might visualise peripheral lesions in patients with PE. Miller et al. (6) (1967) showed that necrosis of lung tissue can be visualised with LUS in patients with PE. However, this procedure has been ignored for years. Nevertheless, with the spread of ultrasound in conjunction with technological advances, clinical studies confirming the original observations have been conducted (1). In a multicentre prospective study, Mathis et al. (7) (2005) showed that detecting pleural-based peripheral lesions and pleural effusions with LUS is useful in the diagnosis of PE.

Bedside LUS can be useful for diagnosis of PE in unstable patients. The literature shows that the wedge sign and pleural effusion seen on LUS are associated with PE (1,2). Our hypothesis is that wedge sign and pleural effusion visualised together or individually but without B-lines may suggest the diagnosis of PE. To our knowledge, there is no study evaluating the role of B-lines in the diagnosis of PE. The aim of this study is to evaluate whether the presence or absence of B-lines contributes to the diagnosis of PE in addition to the wedge sign and pleural effusion.

## MATERIALS AND METHODS

### Study design

The study was conducted as a prospective observational study. Approval from the local ethics committee and the informed consent of all patients participating in the study were obtained.

### Study setting and population

Patients over 18 years old who presented to the Emergency Department (ED) of a university research hospital between 1 April 2013 and 31 June 2014 with complaints of sudden onset of shortness of breath, chest pain, and unexplained syncope and who agreed to participate were included in the study. The exclusion criteria were a history of recent trauma, pregnancy, contrast allergy, renal insufficiency without the need for haemodialysis therapy (glomerular filtration rate <30 mL/min), or refusal to participate in the study.

### Study protocol

All eligible patients were informed about the study. Written informed consent was obtained from the patients who agreed to participate. All of the participants were monitored, and systolic and diastolic blood pressure, respiratory rate, oxygen saturation, and heart rate were recorded. Blood samples were taken for laboratory tests. D-dimer (Ste-LIE TEST, Asnieres Sur Seine, France) and arterial blood gas (ABL 700 series, Copenhagen Radiometer, Denmark) were measured. A level of 0.5 ng/mL was accepted as the cut-off value for D-dimer, as determined by the manufacturer. Electrocardiogram was also obtained for all patients. Demographic data, duration of symptoms, examination findings, comorbid diseases, use of medications, lab results, and Wells scoring were recorded. Low-risk (Wells score ≤4) patients with a negative D-dimer were excluded (8).

Patients participating in the study were first evaluated with bedside LUS, and the findings were recorded on standardised forms. The LUS examinations were performed by only one operator, who had a certificate (Basic Emergency and Procedural Ultrasound Course, 2013) in bedside emergency ultrasound, using an Esaote MyLab Five portable ultrasonography (USG) device with a 1-8 MHz Esaote CA431 curved probe at a depth of 15 cm and a 4-13 MHz Esaote LA523 probe at a depth of 5 cm. Sonographic examinations were performed with the patient in the supine position. To expand the intercostal space, the patients were asked to raise their arms and hold them behind their head. Sonographic examinations were performed through the intercostal spaces on both sides of the thorax at certain points that were predetermined by the BLUE protocol. These points were Blue 1, Blue 2, the phrenic point, and the PLAPS point (9,10). Contrast-enhanced chest CT (Toshiba Aqullio 64, Nai, Japan) was obtained for all participants as a gold standard test with a mean effective radiation dose of 5-7 mSv and 50 mL of non-ionic contrast media. CT images were interpreted and reported by a radiologist. CT results were recorded on the 12-item data collection forms. Management of participants diagnosed with PE was carried out in accordance with the European Society of Cardiology guideline for PE (11).

### Outcome measures

Three predetermined parameters were investigated by sonographic examination performed at each of the specified points. These parameters were B-lines, the wedge sign, and pleural effusion. The presence of three or more B-lines in one area (4) and one or more of the other findings was considered a positive result. Thorax CT angiography was used as a gold standard test for all patients who underwent LUS. According to the tomography reports prepared by radiologists, patients were divided into two groups: with PE or without PE.

### Statistical analysis

Data were analysed with the SPSS software package for Windows, version 21.0 (SPSS Inc., Chicago, IL, USA). The sample size in part A was calculated according to Flahault et al. (12); assuming a 0.05 significance level, n=70 would have 80% power to detect a sensitivity of 90% with a lower acceptable limit of sensitivity of 75%. The sociodemographic and clinical characteristics of patients were expressed as mean ± standard deviation, median, interquartile range, 95% confidence interval, and percentage (%). The student’s t-test was used to compare continuous variables, and the chi-square test was used to compare discrete variables. The Kolmogorov-Smirnov test was used to evaluate whether the data were normally distributed. The Spearman test was used for the correlation coefficients and statistical significance between the ultrasound waves and artefacts, which were ordinal, and the CT angiography findings indicating positive PE. In the multivariate analyses, all of the ultrasound waves and artefacts used as predictors of the diagnosis of PE were examined using an enter method of binary logistic regression analysis. A type 1 error level of less than 5% was considered statistically significant.

## RESULTS

**Figure 1:**
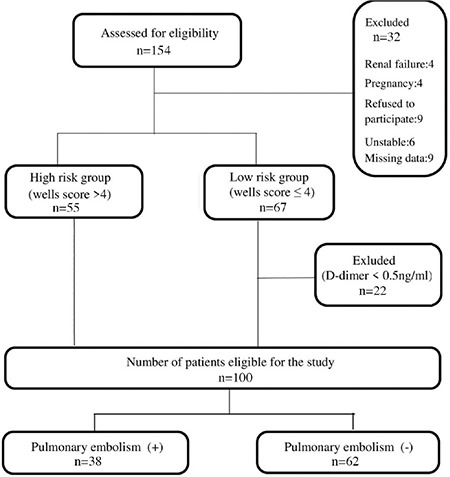
Patient flow chart.

The patient recruitment and flow in the study are shown in Figure 1. The demographics and clinical characteristics of the patients and comparisons between them are shown in Table 1. One hundred patients were included in the study, and 38 (38%) were diagnosed with PE.

We found statistically significant differences between the groups in terms of B-lines and the wedge sign in the comparison of bedside LUS findings (p=0.005 and p<0.001, respectively). We found no significant differences in terms of effusion (p=0.234) (Table 2). Chest CT revealed negative weak correlations with B-lines (r=-0297) and a positive moderate correlation with the wedge sign (r=0.523, p<0.01) (Table 3). The sensitivity, specificity, and positive and negative predictive values of the individual LUS findings were low. In the logistic regression analyses, in which all USG findings were evaluated, the wedge sign (p<0.01, OR=69.45, 95% CI=6.94-695.17) and B-lines (p=0.033, OR=1.96, 95% CI=0.41-8.40) were found to be effective in the diagnosis of PE (Table 4).

## DISCUSSION

We found that none of the individual findings had sufficient diagnostic value for the diagnosis of PE. Although the sensitivity and specificity of the findings were low, the presence of the wedge sign and the absence of B-lines were found to be valuable in the diagnosis of PE.

Although many studies have evaluated B-lines, to our knowledge, none have investigated the value of B-lines in the diagnosis of PE. B-lines are useful for detecting interstitial lung disease (13), and it has been reported that the B-lines have 85.7% sensitivity and 97.7% specificity in defining alveolar interstitial syndrome (14). Lichtenstein and Mezière (15) showed that the B-lines had a sensitivity of 100% and a specificity of 92% in indicating pulmonary oedema, and they suggested that LUS might be used in differentiating pulmonary oedema from COPD. An increased number of B-lines is typical for sonographic imaging of pulmonary oedema. Based on this, we hypothesise that B-lines might not be seen due to decreased perfusion of lung tissue in early PE. In this study, we observed a negative correlation between the B-lines and PE. This suggests that LUS results in which the wedge sign is visualised but the B-lines are not can be interpreted in favour of PE. The visualisation of B-lines together with the wedge sign might indicate conditions other than PE. However, these results can be misleading due to the small number of patients studied; there is a need for further studies including more patients.

The role of LUS has traditionally limited its utility to detect pulmonary disease. However, in recent years it has become a tool to complement conventional radiography in a variety of pulmonary, mediastinal, and pleural diseases (1). For example, LUS was reported to be an effective, fast, and accurate tool in the diagnosis of pneumothorax (16). It has also been used in the evaluation of pneumonia (17) and pleural effusion (18). Lichtenstein and Mezière (4) developed a new perspective, which they referred to as the BLUE protocol, for diagnosing severe pathologies that cause shortness of breath, such as pneumonia, PE, COPD, asthma, pulmonary oedema, and pneumothorax.

Studies investigating the use of LUS in the diagnosis of PE first emerged with the introduction of defining peripheral lesions by ultrasound images in the late 1960s. In their prospective study conducted on 352 patients, Mathis et al. (7) observed wedge and round-shaped pleural-based peripheral lesions, and they found that the sensitivity of these lesions for the diagnosis of PE was 74%, the specificity was 95%, the positive predictive value was 95%, and the negative predictive value was 75%. They reported that these findings were valuable in the diagnosis of PE, but negative results did not exclude such a diagnosis.

Over the following years, numerous clinical studies were conducted on this subject (19,20,21). Thromboembolic occlusion of a pulmonary artery causes decreased synthesis of surfactant in the affected area, increased pulmonary vascular resistance, and increased capillary permeability (22). When the alveoli are collapsed and filled with fluid, USG waves are allowed to penetrate deeper into the lung parenchyma, creating an acoustic window. Due to impaired perfusion, PE may also lead to a wedge-shaped pleural-based pulmonary infarction (23). This oedematous infarction area can be seen on ultrasound as the wedge sign. However, pulmonary infarction is not a common situation, because the circulation to the lungs arises from the bronchial, as well as the pulmonary, arteries. In this study, we found that although the sensitivity and specificity were low, the wedge sign was valuable in the diagnosis of PE. The patients in this study were evaluated within a few hours of the onset of symptoms, and thus oedema, alveolar haemorrhage, and tissue necrosis had not yet occurred by the time we performed LUS. This may explain why our results were different from those of other studies.

This was a single-centre prospective study carried out in an ED setting. Under these conditions, many confounding factors may have been overlooked. We believe that the power of this single-centre study is low due to the sample size of PE diagnoses; since a single researcher conducted the LUS examinations, the study only included patients who were admitted during his time on duty in the ED, which had a negative impact on the sample size. In addition, we did not compare the LUS findings according to the duration of symptoms. A long duration of ischemic state causes necrosis of the lung parenchyma, and these necrotic areas may have a different appearance from normal tissue on LUS.

## Figures and Tables

**Table 1:**
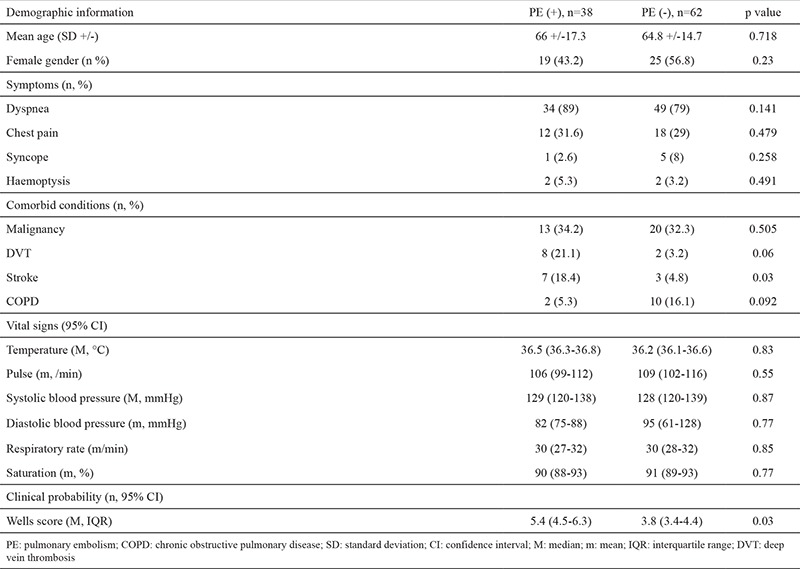
Demographic and clinical information

**Table 2:**
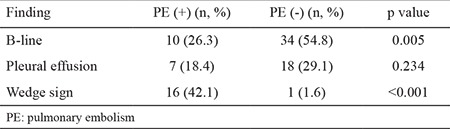
Distribution of ultrasound findings between groups

**Table 3:**
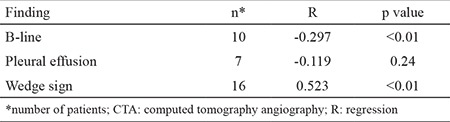
Correlation between CTA results and ultrasonographic findings

**Table 4:**
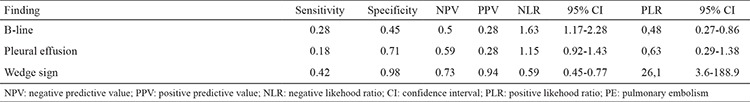
Test performance of B-line, pleural effusion, and wedge sign on 38 patients with PE
